# Right Paraduodenal Hernia as a Cause of Acute Abdominal Pain in the Emergency Department: A Case Report and Review of the Literature

**DOI:** 10.3390/diagnostics12112742

**Published:** 2022-11-09

**Authors:** Viktoria Lamprou, Despoina Krokou, Eleni Karlafti, Stavros Panidis, Leonidas Kougias, Georgios Tzikos, Aristeidis Ioannidis, Smaro Netta, Evanthia Thomaidou, Daniel Paramythiotis

**Affiliations:** 1 Radiology Department, University General Hospital of Thessaloniki AHEPA, 54636 Thessaloniki, Greece; 2 First Propaedeutic Surgery Department, University General Hospital of Thessaloniki AHEPA, 54636 Thessaloniki, Greece; 3 Emergency Department, University General Hospital of Thessaloniki AHEPA, Aristotle University of Thessaloniki, 54636 Thessaloniki, Greece; 4 Department of Interventional Radiology, University General Hospital of Thessaloniki AHEPA, 54636 Thessaloniki, Greece; 5 Department of Anesthesia and Intensive Care, University General Hospital of Thessaloniki AHEPA, 54636 Thessaloniki, Greece

**Keywords:** acute abdomen, internal hernia, bowel malrotation, bowel obstruction, laparoscopy, laparotomy

## Abstract

Paraduodenal hernias (PDHs) represent an unusual cause of acute abdominal pain in the Emergency Department (ED) and are associated with high morbidity attributable to a challenging clinical and radiological diagnosis, as signs and symptoms mimic other frequent causes of acute abdominal pain. We report a right paraduodenal hernia in a 37-year-old female patient who presented to the ED complaining of abdominal pain located in the right lower abdomen and hypogastrium, accompanied by nausea. During diagnostic work up, the abdominal computed tomography scan revealed the presence of small bowel malrotation with concomitant right paraduodenal hernia. These findings were confirmed intraoperatively. We performed a brief literature review about the clinical manifestations and treatment options of right paraduodenal hernias, which retrieved only 30 articles related to this condition. Prompt diagnosis, radiological or intraoperative, of paraduodenal hernias is crucial because nearly 50% will progress to small bowel obstruction. Therefore, it is essential for every clinician to account for them in the differential diagnosis of acute abdominal pain in the ED.

## 1. Introduction

An internal intestinal hernia is a rarely encountered condition, with an incidence of less than 1%, and develops when bowel loops penetrate through a peritoneal or mesenteric defect into the abdominal compartment [1]. It is associated with high mortality rates, ranging from 20% to 75% if bowel obstruction and strangulation are present [2]. Fifty-three percent of internal abdominal herniations occur at the paraduodenal recess [3]. Two variants of paraduodenal herniations are described to have clinical and surgical importance: left-sided, in which the small intestine prolapses through the Landzert’s fossa, and right-sided, in which bowel herniation occurs through the Waldeyer’s fossa [3,4]. Right-sided paraduodenal hernia is rarer than its counterpart, ranging up to 25% of cases [4]. Symptoms of internal abdominal herniations depend on the reducibility of the herniated bowel loops varying from vague epigastric pain in cases of spontaneous reduction to severe colicky periumbilical pain in cases of incarceration and bowel ischemia [5]. This variability of symptoms may overlap with other causes of acute abdominal pain in the emergency department, making paraduodenal hernias one of the challenging diagnostic entities for clinicians. In such cases, a high index of suspicion is required and an accurate diagnosis may be established by a computed tomography (CT) scan [6,7]. It is essential to timely diagnose and restore paraduodenal hernias, whether they are symptomatic or not because, if left untreated, they can progress to acute small bowel obstruction, ischemia, and bowel perforation [8].

## 2. Case Presentation

A 37-year-old female presented to the emergency department with complaints of acute hypogastric pain, accompanied by nausea, which persisted for 6 h prior to presentation. She had no history of abdominal surgery nor trauma or known morbidities, but reported occasional postprandial abdominal pain.

The clinical examination revealed abdominal tenderness localized in the right lower abdomen and hypogastrium, positive McBurney’s sign, and rebound tenderness. Bowel sounds were normal and no abdominal distention was noted. Laboratory results were unremarkable except for elevated leucocyte count, which was 19,000/μL. Based on clinical presentation and physical examination, differential diagnoses included acute appendicitis, gynecological disorders, and inflammatory bowel disease.

Initial imaging work-up included abdominal X-ray and ultrasonography with findings negative for gynecological causes of abdominal pain, but inconclusive of acute appendicitis.

**Figure 1:**
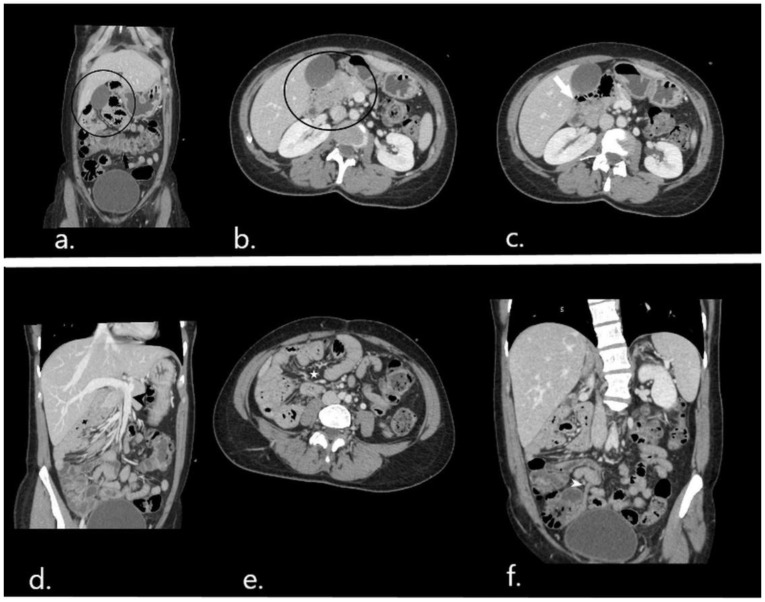
CT scan on admission: coronal (**a**) and axial (**b**) images depict the abnormal position of small bowel loops (in black circle) above the transverse colon (black arrow) and to the right. On axial images (**c**) the horizontal part of the duodenum (white arrow) is positioned on the right and does not run to the left ventrally from the abdominal aorta and inferior vena cava. (**d**) Maximum intensity projection (MIP) image reconstruction on the coronal plane shows an abnormal left course of the superior mesenteric vein (black arrowhead). (**e**) Mesenteric vessel congestion at the site of small bowel herniation. (**f**) Coronal reformatted image showing a normal appendix (white arrowhead).

In this setting, a computed tomography (CT) scan of the abdomen and pelvis was performed. The protocol included thin (0.625 mm) slices before and after intravenous contrast administration, and images were then reformatted in the axial, coronal, and sagittal planes. Oral contrast to opacify the gastrointestinal lumen was not used as this case was considered an emergency. CT scanning demonstrated an abnormally low position of the transverse colon and right colic flexure with the concomitant presence of small bowel loops above and to the right (Figure 1a,b). The third part of the duodenum could not be visualized in its expected position (Figure 1c). There was an abnormal superior mesenteric artery (SMA)–superior mesenteric vein (SMV) relationship with the vein coursing on the left side of the artery (Figure 1d). These imaging findings highly suggested the presence of intestinal malrotation. Moreover, the cluster of the small bowel loops situated above and to the right of the transverse colon showed evidence of mesenteric congestion with prominent vessels but no signs of bowel ischemia (Figure 1e). The appendix did not show any signs of inflammation (Figure 1f). The overall imaging findings, associated with the clinical status, implied the presence of a right paraduodenal internal herniation as a complication of intestinal malrotation.

**Figure 2:**
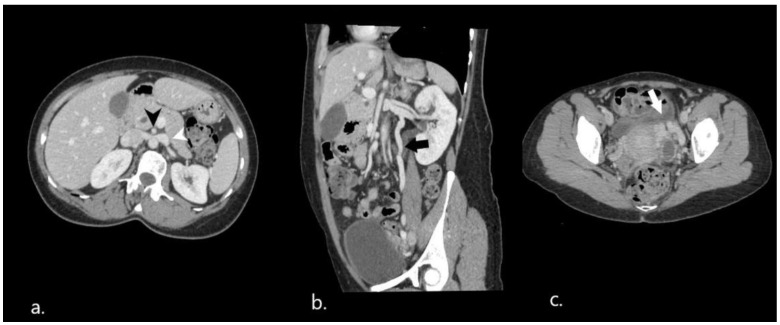
Nutcracker phenomenon on initial CT scan. (**a**) On axial images, the hilar portion of the left renal vein (white arrowhead) is distended as a result of the vein compression between the aorta and the superior mesenteric artery (black arrowhead) (**b**). Coronal reformatted image showing engorged left gonadal vein (black arrow). (**c**) Accompanying pelvic varices.

Another finding, which was considered to be incidental, was the formation of an acute angle between the aorta and superior mesenteric artery, which resulted in compression of the left renal vein (Figure 2a), an entity known as the nutcracker phenomenon. The left ovarian vein was abnormally distended (Figure 2b) and multiple pelvic venous varices were identified (Figure 2c).

**Figure 3:**
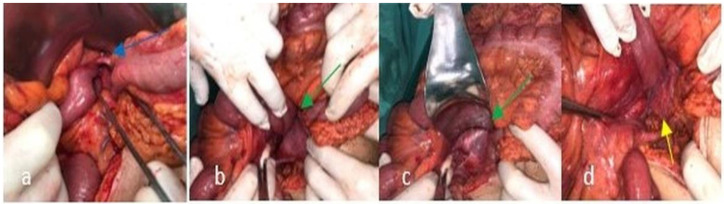
Perioperative findings: (**a**) small intestine loops on the right of Treitz’s ligament (blue arrow). (**b**,**c**). The orifice of the hernia (green arrow). (**d**) Repositioned hernia and closure of the gap (yellow arrow).

The patient was managed conservatively with intravenous fluids and antibiotics under close clinical and laboratory observation, as there were no signs of complete ileus or bowel ischemia. The patient’s clinical condition improved over time during conservative treatment, while nausea and abdominal pain decreased, probably because of spontaneous retraction of small bowel herniation. However, 72 h later, the patient deteriorated progressively with increasing abdominal pain and vomiting. Surgery was then decided. Under general anesthesia, a limited midline sub-umbilical incision was made, the white line was opened, and an entrance to the peritoneal cavity was made, and pneumoperitoneum was created (open Hasson technique). Due to the presence of dilated small bowel loops accompanied by hemorrhagic and dirty fluid in the peritoneal cavity, the laparoscopy was converted to an open laparotomy. The hernia sac was identified on the right side of Treitz’s ligament, containing small intestine loops (Figure 3a). Apparently, a congenital gap near Treitz’s ligament formed the orifice of the internal hernia, a fact that confirmed the diagnosis of a right paraduodenal hernia (Figure 3b,c). The hernia was repositioned, and the gap was closed using Vicryl 3/0 sutures (Figure 3d). After hernia reduction, it was obvious that the third part of the duodenum and the duodenojejunal flexure were positioned on the right side of the abdomen along with normal small bowel loops, confirming the radiological findings of bowel malrotation. An appendectomy was also performed. The postoperative period was uneventful, and the patient was discharged 5 days later. 

**Figure 4:**
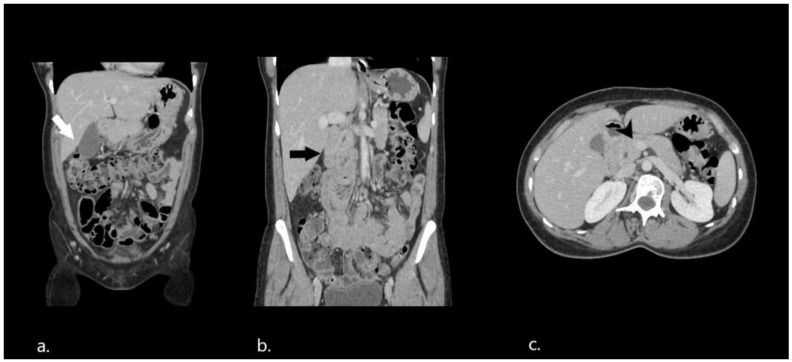
Postoperative abdominal CT. (**a**,**b**) Coronal reformatted images depicting no small bowel loops located above and to the right of the transverse colon (white arrow) and malrotated duodenum and jejunum loops on the right side of the abdomen (black arrow). (**c**) Restoration of the normal course of the superior mesenteric vein (black arrowhead).

One month after surgery, a follow-up CT scan was performed with an identical scanning protocol to the previous one (Figure 4). There were no intestinal loops located in an abnormal position above and to the right of the transverse colon. No evidence of mesenteric congestion or fat infiltration was noticed. The normal relationship between the superior mesenteric vessels was restored, however, the third part of the duodenum and the duodenojejunal flexure remained on the right side of the abdomen and did not cross the midline (Figure 4b) No changes were noticed regarding the left renal vein compression. The patient did not experience any new symptoms during the follow-up.

## 3. Discussion

Paraduodenal hernias (PDH) are the most frequently encountered types of internal hernias [4]. They are more prevalent in males, with a 2:1 ratio, and may be detected at any age. Two variants of PDH are of significant clinical importance, the right-sided, in which the small bowel is herniated through the fossa of Waldeyer, and the left-sided, in which herniation occurs through the fossa of Landzert, with the latter being more common [8]. PDHs usually develop during the embryonic period as a consequence of abnormal rotation and fixation of the midgut. As a result, they represent a congenital anatomical anomaly associated with intestinal tract malrotation, however, internal hernias can also occur after abdominal traumas, infections, or previous abdominal surgery [4,6,9]. During the fetal time, the midgut passes through an orderly pattern of rotation which is divided into three stages. An abnormality during the second stage of this pattern results in the development of small bowel malrotation and paraduodenal hernia. In that time, the midgut returns from the yolk sac to the abdominal cavity and the small bowel is situated on the right side of the abdomen as it has already rotated 90 degrees counterclockwise. In the normal sequence of events, the small bowel should perform an additional 180°counterclockwise rotation and take its normal position behind and to the left of the superior mesenteric artery. If this additional rotation fails to happen, oneportion of the small bowel remains on the right of the superior mesenteric artery and confines in a hernial sac behind the colonic mesentery forming the right type of paraduodenal hernia [10,11,12].

Clinical manifestations of right PDH include diverse non-specific symptoms, varying from acute to chronic abdominal pain accompanied by nausea and vomiting, and less commonly, fever. Patients may report a history of recurrent ileus since childhood, weight loss, or may be asymptomatic [5]. In our case, symptoms and physical findings were consistent with more common causes of acute abdominal pain, such as acute appendicitis, and only the employment of abdominal CT led to the correct diagnosis. In fact, multidetector CT is the imaging modality of choice for the diagnosis and preoperative evaluation of internal hernias [6,7]. On the CT scan, the right paraduodenal hernia typically appears as an abnormal aggregation of dilated small bowel loops in the form of a saclike mass within the fossa of Waldeyer at the right upper abdomen. The third portion of the duodenum lies superiorly to the dilated loops while the root of the small bowel mesentery anteriorly. The superior mesenteric vessels demarcate the anteromedial margin of the fossa and the right colic vein is displaced anteriorly [3,6,9]. If intestinal malrotation coexists, the normal third portion of the duodenum is not visualized crossing from right to left, and the superior mesenteric vein appears on the left in relation to the superior mesenteric artery [11,12]. When the hernia is complicated by obstruction, associated radiologic findings such as small bowel loop dilatation, air-fluid levels, mesenteric congestion, and fat stranding are present. In cases of strangulated bowel obstruction, CT findings include reduced mural enhancement and free peritoneal fluid [13]. Additionally, abdominal CT can exclude other causes of abdominal pain or depict concomitant conditions. A rare association that has been recently highlighted by a few case reports is that between gut rotation abnormalities and the nutcracker phenomenon [14,15,16]. The nutcracker phenomenon involves the entrapment and compression of the left renal vein between the aorta and SMA when the angle they form is steeper than usual. If this condition becomes symptomatic, the term nutcracker syndrome is used and is characterized by hematuria, orthostatic proteinuria, abdominal or flank pain, gonadal vein syndrome, and varicocele [17]. In the present case, no symptoms of renal involvement were present and surgical treatment was not considered.

Although intestinal obstruction occurs in only 1% of all cases of internal hernias, nearly 50% of all patients with PDH will develop acute bowel obstruction with a mortality rate of up to 50% [2,18]. Thus, an early surgical repair is of crucial importance after diagnosis. The standard surgical approach is a laparoscopic repair because of better postoperative outcomes compared to open surgery [19]. During the surgical correction of a right paraduodenal hernia, the small intestine is restored to the site that it would normally be after the end of the first stage of intestinal rotation, followed by either closure of the defect or extension of the hernial orifice. However, opening the hernia sac should be avoided because the superior mesenteric vessels lie in close proximity to the hernial orifice, and opening it directly may injure the blood supply to the intestine or cause massive blood loss [8]. Open conversion of laparoscopic management may be needed when severe bowel distention is present preoperatively (bowel diameter > 4cm) and if bowel necrosis is found [20]. In the present case, we believe that initial conservative treatment alleviated the patient’s symptoms due to the spontaneous reduction of herniated bowel loops, but in accordance with previously reported cases by Mehra et al. [8] and Lin et al. [21], bowel obstruction reoccurred within 72 h and surgery was mandatory. This highlights the importance of timely surgical intervention, as bowel obstruction may occur at any point in time even if the patient shows clinical improvement.

We performed a literature search on PubMed database using the term “right paraduodenal hernia”. The articles retrieved were in English and no other search restrictions were applied. In the current literature, 32 cases of right paraduodenal hernias have been reported (Table 1). The vast majority of patients present with diffuse abdominal pain accompanied by vomiting or nausea or signs of acute small bowel obstruction. In all cases, an investigation surgery, open or laparoscopic, was conducted. The overall outcome was complete recovery of the patient.

## 4. Conclusions

Right paraduodenal hernias are a rare abdominal condition with varying manifestations ranging from incidental findings to acute bowel obstruction with high mortality. A CT scan is an important diagnostic tool in acute and chronic abdominal symptoms associated with PHDs and should be obtained immediately. Early and correct diagnosis is essential, as only surgical treatment prevents intestinal complications.

## Figures and Tables

**Table 1:** Previously reported cases of right PDHs.

Authors	Gender	Age	Symptoms	Surgical Method	Outcome
S.Manfredelli et al. [4]	F	86	Acute bowel obstruction syndrome	Exploratory laparotomy	C/R
R. Mehra et al. [8]	M M	46 23	Nausea/vomit Abdominal pain and bilious vomit	Exploratory laparotomy	C/R
CT. Lin et al. [21]	M	30	Abdominal pain Nausea/vomit	Exploratory laparotomy	C/R
B. Kwan et al. [22]	F	18	Subacute small bowel obstruction	Biosynthetic reinforcement	C/R
V.A. Ismavel et al. [23]	M	23	Abdominal pain Nausea/vomit	Exploratory laparotomy	C/R
J.G Bittner et al. [24]	F	26	Abdominal pain	Laparoscopy	C/R
N. Poudel et al. [25]	M	36	Abdominal pain	Exploratory laparotomy	C/R
J.M.Manipadam et al. [26]	F	31	Abdominal pain Occasional vomit	Exploratory laparoscopy	C/R
S. Walkner et al. [27]	M	37	Abdominal pain Nausea/vomit	Exploratory laparoscopy	C/R
K. Shadhu et al. [28]	M	40	Abdominal pain	Exploratory laparoscopy	C / R
M. Hassan et al. [29]	M	19	Abdominal pain	Exploratory laparotomy	C/R
M. Ong et al. [30]	F	53	Abdominal pain Constipation Tenesmus	Exploratory laparotomy	C/R
K. Bharatam et al. [31]	M	30	Abdominal pain	Exploratory laparotomy	C/R
N. Omarov et al. [32]	M	59	Abdominal pain	Exploratory laparoscopy	C/R
A.M. Joseph et al. [33]	M	43	Abdominal pain Nausea/vomit	Laparoscopy converted to laparotomy	C/R
K. Oshita et al. [34]	M	30	Abdominal pain Nausea/vomit	Exploratory laparoscopy	C/R
AR. Bollampally et al. [35]	M	29	Abdominal pain Bilious vomit	Exploratory laparotomy	C/R
JH. Cho et al. [36]	M	30	Abdominal pain Nausea/vomit	Exploratory laparotomy	C/R
E.Antedomenico et al. [37]	F	24	Abdominal pain Nausea/vomit	Exploratory laparoscopy	C/R
T. Tomino et al. [38]	M	23	Abdominal pain Nausea/vomit	Exploratory laparoscopy	C/R
T. Fukada et al. [39]	M	46	Abdominal pain	Exploratory laparotomy	C/R
CM. Nuño-Guzmán et al. [40]	M	41	Intestinal obstruction	Exploratory laparotomy	C/R
CW. Lu et al. [41]	F	45	Periumbilical pain	Exploratory laparotomy	C/R
V. Indiran et al. [42]	M	19	Abdominal pain Nausea/vomit Constipation	Exploratory laparotomy	C/R
A. Martín-Lagos-Maldonado et al. [43]	M	52	Periumbilical pain Nausea/vomit	Exploratory laparotomy	C/R
E. Erdas et al. [44]	F	32	Abdominal pain Nausea/vomit	Exploratory laparoscopy	C/R
A. Abdullah et al. [45]	F	48	Abdominal pain Distention	Exploratory laparotomy	C/R
WC. Brunner et al. [46]	M	60	Constipation Abdominal pain	Exploratory laparoscopy	C/R
T. Takagishi et al. [47]	F (4) M(4)	23–80	Abdominal pain (8) Nausea/vomit (6)	Laparoscopy (7) Laparotomy migration (1)	C/R (6) Postoperative conservative treatment of postoperative ileus (1) Second operation performed (1)
S. McCain et al. [48]	F	41	Abdominal pain Distention Feculent vomit	Exploratory laparotomy	C/R
